# Supplementary material for: Mechanism-Based Approach to New Antibiotic Producers Screening among Actinomycetes in the Course of the Citizen Science Project
Source: Antibiotics (Basel). 2022 Sep 5;11(9):1198. doi: 10.3390/antibiotics11091198 (PMC9495171; doi:10.3390/antibiotics11091198)
Supplement: Supplementary file 1 [file antibiotics-11-01198-s001.zip › Supplementary Materials.pdf]

antibiotics

Supplementary Materials

Mechanism-Based Approach to New Antibiotic Producers Screening among Actinomycetes in the Course of the Citizen Science Project

Inna A. Volynkina [1,2,*], Yuliya V. Zakalyukina [3,4], Vera A. Alferova [5,6], Albina R. Belik [3], Daria K. Yagoda [7], Arina A. Nikandrova [1], Yuliya A. Buyuklyan [3], Andrei V. Udalov [3], Evgenii V. Golovin [3], Maxim A. Kryakvin [7], Dmitrii A. Lukianov [1,2], Mikhail V. Biryukov [1,3,8], Petr V. Sergiev [1,2], Olga A. Dontsova [1,2,5] and Ilya A. Osterman [1,2,3,*]

[1] Center of Life Sciences, Skolkovo Institute of Science and Technology, Bolshoy Boulevard 30, bld. 1, 121205 Moscow, Russia
[2] Department of Chemistry, Lomonosov Moscow State University, Leninskie Gory 1, 119991 Moscow, Russia
[3] Center for Translational Medicine, Sirius University of Science and Technology, Olympic Avenue 1, 354340 Sochi, Russia
[4] Department of Soil Science, Lomonosov Moscow State University, Leninskie Gory 1, 119991 Moscow, Russia
[5] Shemyakin-Ovchinnikov Institute of Bioorganic Chemistry, Miklukho-Maklaya 16/10, 117997 Moscow, Russia
[6] Gause Institute of New Antibiotics, B. Pirogovskaya 11, 119021 Moscow, Russia
[7] School of Bioengineering and Bioinformatics, Lomonosov Moscow State University, Leninskie Gory 1, 119991 Moscow, Russia
[8] Department of Biology, Lomonosov Moscow State University, Leninskie Gory 1, 119991 Moscow, Russia
* Correspondence: Inna.Volynkina@skoltech.ru (I.A.V.); i.osterman@skoltech.ru (I.A.O.)

antibiotics

Contents

Figure S1. Double reporter system pDualrep3 1

Figure S2. Agar block diffusion assay of isolated actinomycetes (BV113, KB-1, Nm4) 2

Figure S3. Utilization of different sugars (1.0%, w/v) as sole carbon source by strain KB-1 3

Figure S4. Utilization of different sugars (1.0%, w/v) as sole carbon source by strain BV113 4

Figure S5. MS/MS spectrum of the pikromycin $[M+H]^+$ ion at *m/z* value 526.3370 5

Figure S6. MS/MS spectrum of the chartreusin $[M+Na]^+$ ion at *m/z* value 663.1677 6

Table S1. Physiological and biochemical characteristics of KB-1 isolate and closely related *Streptomyces* sp. strains 7

Table S2. Physiological and biochemical characteristics of BV113 isolate and closely related *Streptomyces* sp. strains 9

Table S3. Composition of some nutrient media used 11

Supplementary References 13

Figure S1. Double reporter system pDualrep3

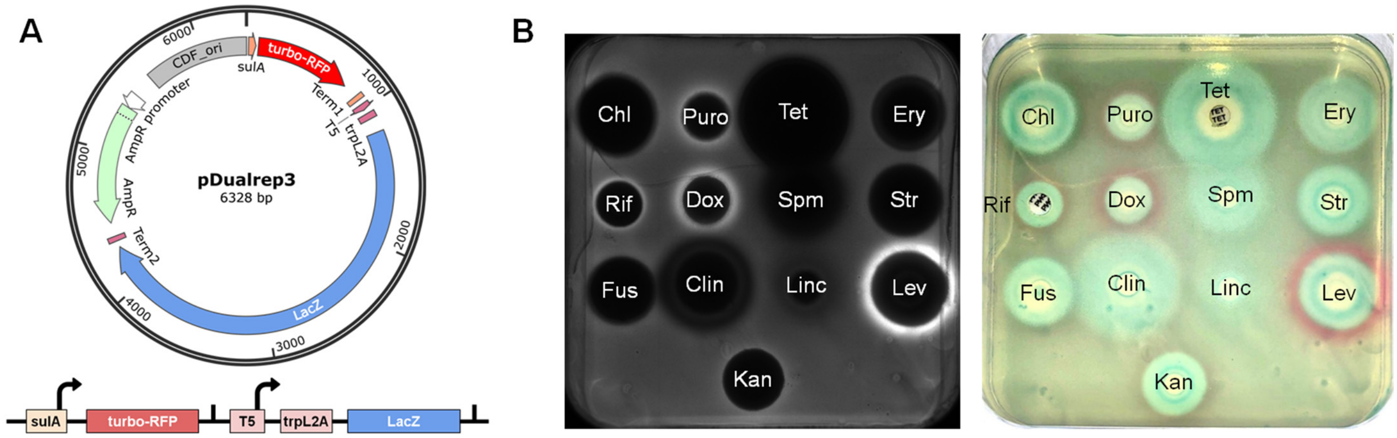

(**A**) pDualrep3 plasmid map and the reporter scheme. CDF_ori, CloDF13-derived CDF replicon; sulA, promoter of the *sulA* gene; turbo-RFP, red fluorescent protein gene; T5, bacteriophage T5 promoter; trpL2A, modified *trpL* leader open reading frame carrying W10A and W11A substitutions; LacZ, β-galactosidase gene. Transcription start sites are shown by arrows. Transcription terminators are shown by vertical dashes. (**B**) Comparison of fluorescence signals using an imaging system (left) and with the naked eye (right). The plate was scanned in the Cy3 (for TurboRFP) channel. *Turbo-rfp* expression is visible in red to the naked eye in the zone of antibiotic sublethal concentrations. *LacZ* expression is visible in blue to the naked eye in the zone of antibiotic sublethal concentrations. An agar plate was coated with the *E.coli ΔtolC* strain transformed with the pDualrep3 plasmid. A panel of antibiotics was used: chloramphenicol (Chl), puromycin (Puro), tetracycline (Tet), erythromycin (Ery), rifampicin (Rif), doxorubicin (Dox), spectinomycin (Spm), streptomycin (Str), fusidic acid (Fus), clindamycin (Clin), lincomycin (Linc), levofloxacin (Lev) and kanamycin (Kan).

Figure S2. Agar block diffusion assay of isolated actinomycetes (BV113, KB-1, Nm4)

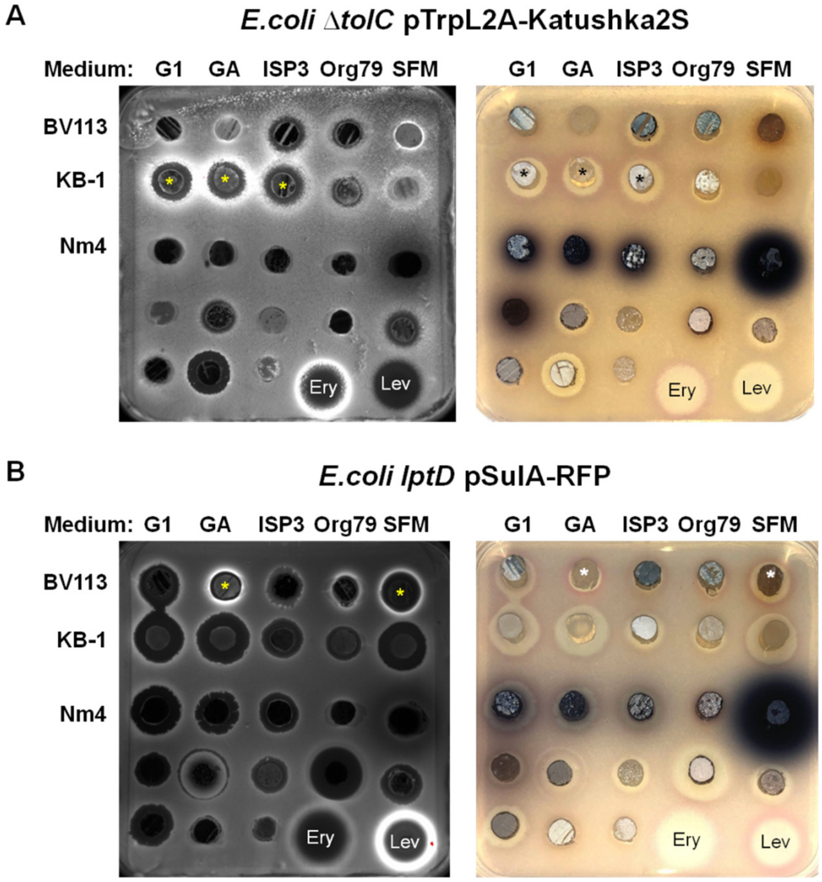

Different nutrient media (G1, GA, ISP3, Org79, SFM) were used to obtain blocks with mycelium and assess their antibacterial activity (for details, see Table S3). *Streptomyces* sp. KB-1 produces a compound that inhibits the growth of *E.coli ΔtolC* and *lptD* strains, revealing a strong pTrpL2A-Katushka2S reporter induction (marked with asterisks (*) in the picture **A**). *Streptomyces* sp. BV113 produces a compound that primarily inhibits the growth of *E.coli lptD* strain, revealing a strong pSulA-RFP reporter induction (marked with asterisks (*) in the picture **B**). Comparison of fluorescence signals using an imaging system (left) and with the naked eye (right). The upper plate (**A**) was scanned in the Cy5 (for Katushka2S) channel, the lower plate (**B**) was scanned in the Cy3 (TurboRFP) channel (left). Katushka2S and TurboRFP are visible to the naked eye in lilac and orange-red colors, respectively, in the zone of antibiotic sublethal concentrations (right). Agar plates were coated with either the *E.coli ΔtolC* strain transformed with the pTrpL2A-Katushka2S plasmid, or the *E.coli lptD* strain transformed with the pSulA-RFP plasmid. Antibiotics erythromycin (Ery) and levofloxacin (Lev) were used as controls.

Figure S3. Utilization of different sugars (1.0%, w/v) as sole carbon source by strain KB-1

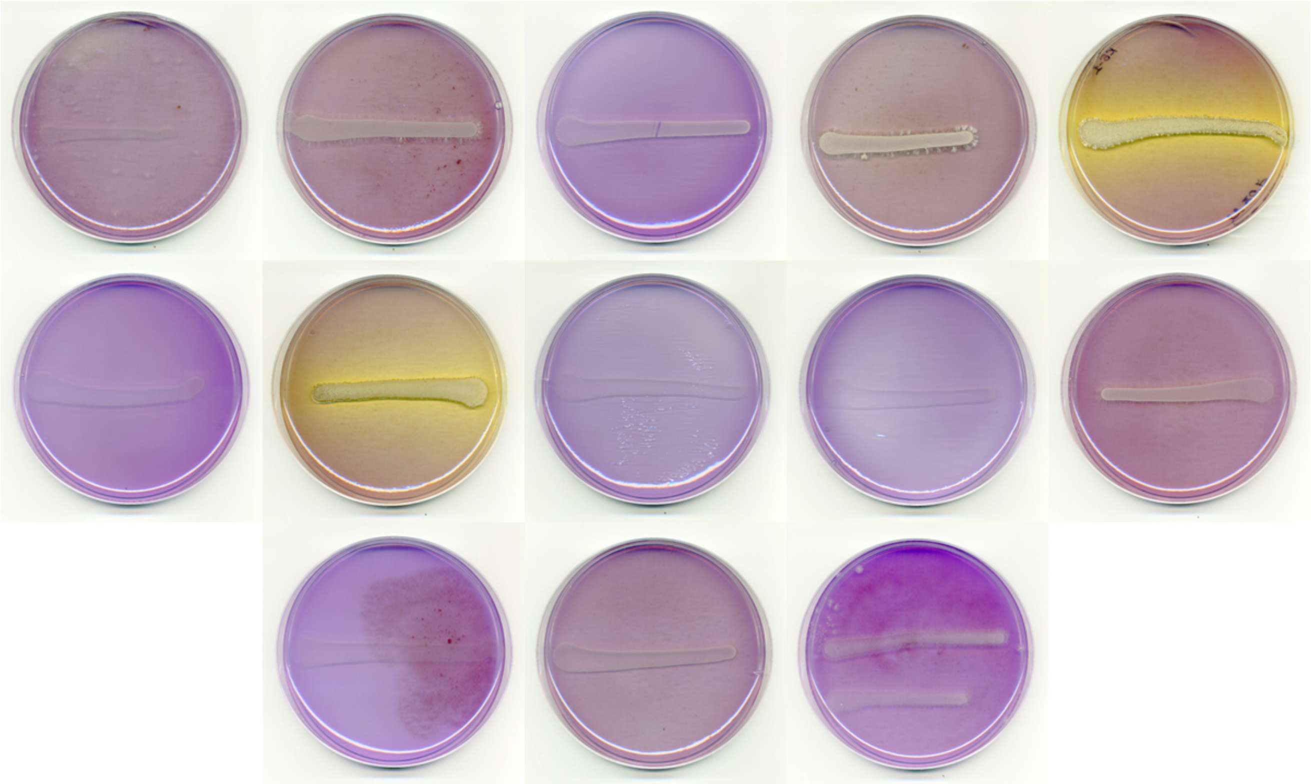

Top row: basal agar medium (ISP9) without sugar, arabinose, fructose, galactose, glucose. Middle row: inositol, maltose, mannite, raffinose, rhamnose. Bottom row: sorbitol, sucrose, xylose.

Figure S4. Utilization of different sugars (1.0%, w/v) as sole carbon source by strain BV113

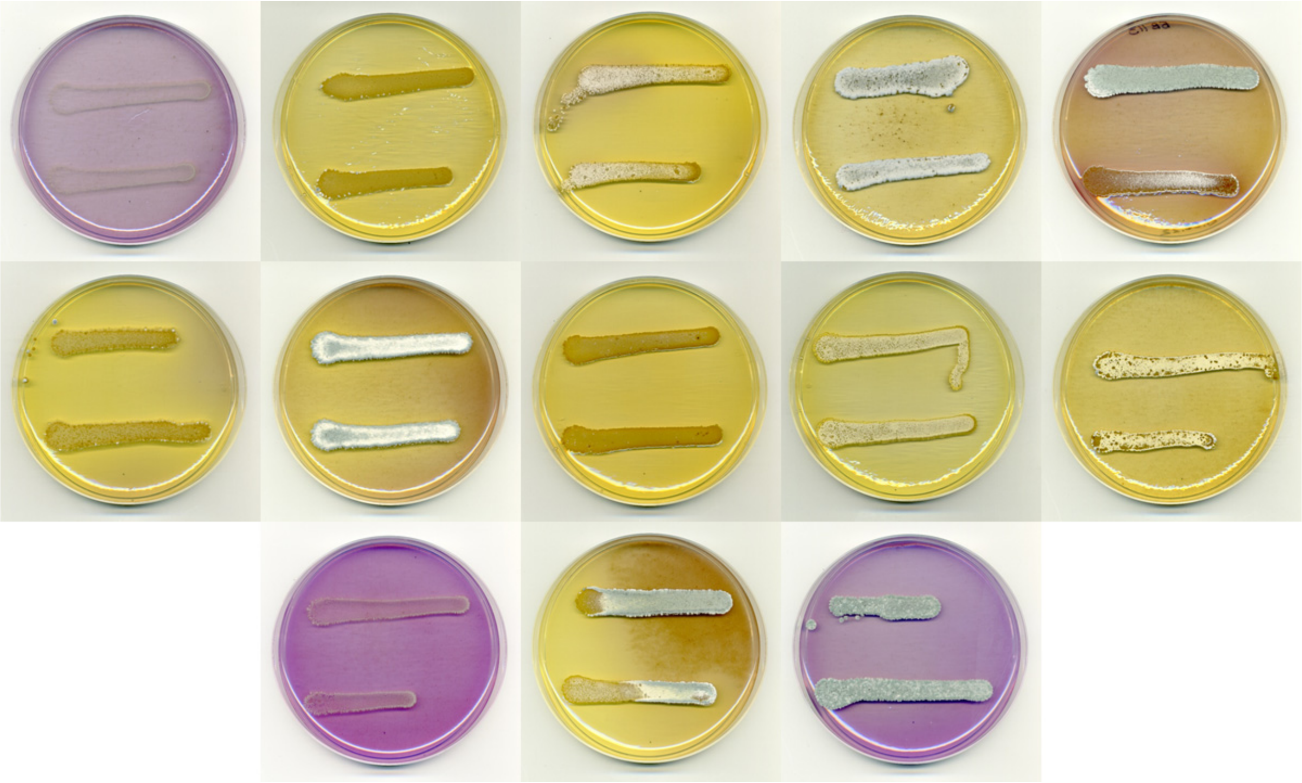

Top row: basal agar medium (ISP9) without sugar, arabinose, fructose, galactose, glucose. Middle row: inositol, maltose, mannite, raffinose, rhamnose. Bottom row: sorbitol, sucrose, xylose.

Figure S5. MS/MS spectrum of the pikromycin $[M+H]^+$ ion at *m/z* value 526.3370

Generic Display Report

Analysis Info

Analysis Name	D:\Data\OS\OS_KB1_F3_6_RA3_1_31.d	Acquisition Date	8/2/2021 12:55:10 PM
Method	10_95ACN_HE_MSMS.m	Operator	Demo User
Sample Name	OS_KB1_F3_6	Instrument	maXis II ETD
Comment			

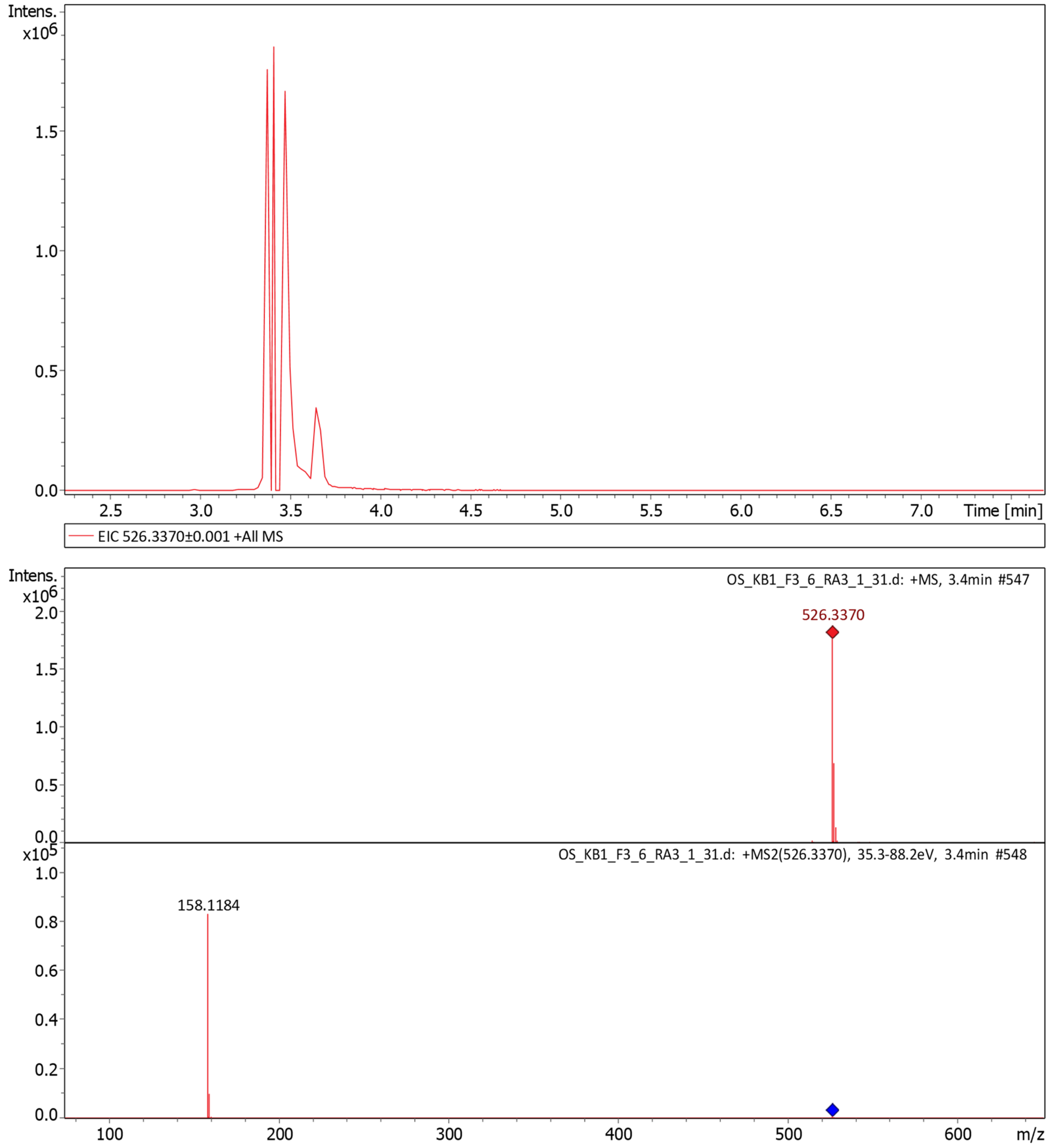

Bruker Compass DataAnalysis 5.2 printed: 8/24/2022 11:43:01 AM by: demo Page 1 of 1

Figure S6. MS/MS spectrum of the chartreusin $[M+Na]^+$ ion at *m/z* value 663.1677

Generic Display Report

**Analysis Info**		Acquisition Date	7/20/2021 4:54:22 PM
Analysis Name	D:\Data\Service\210621\OS_BV113-ACN30_8_9.d		
Method	Syringe_MSMS_POS.m	Operator	Demo User
Sample Name	OS_BV113-ACN30_8_9	Instrument	maXis II ETD
Comment			

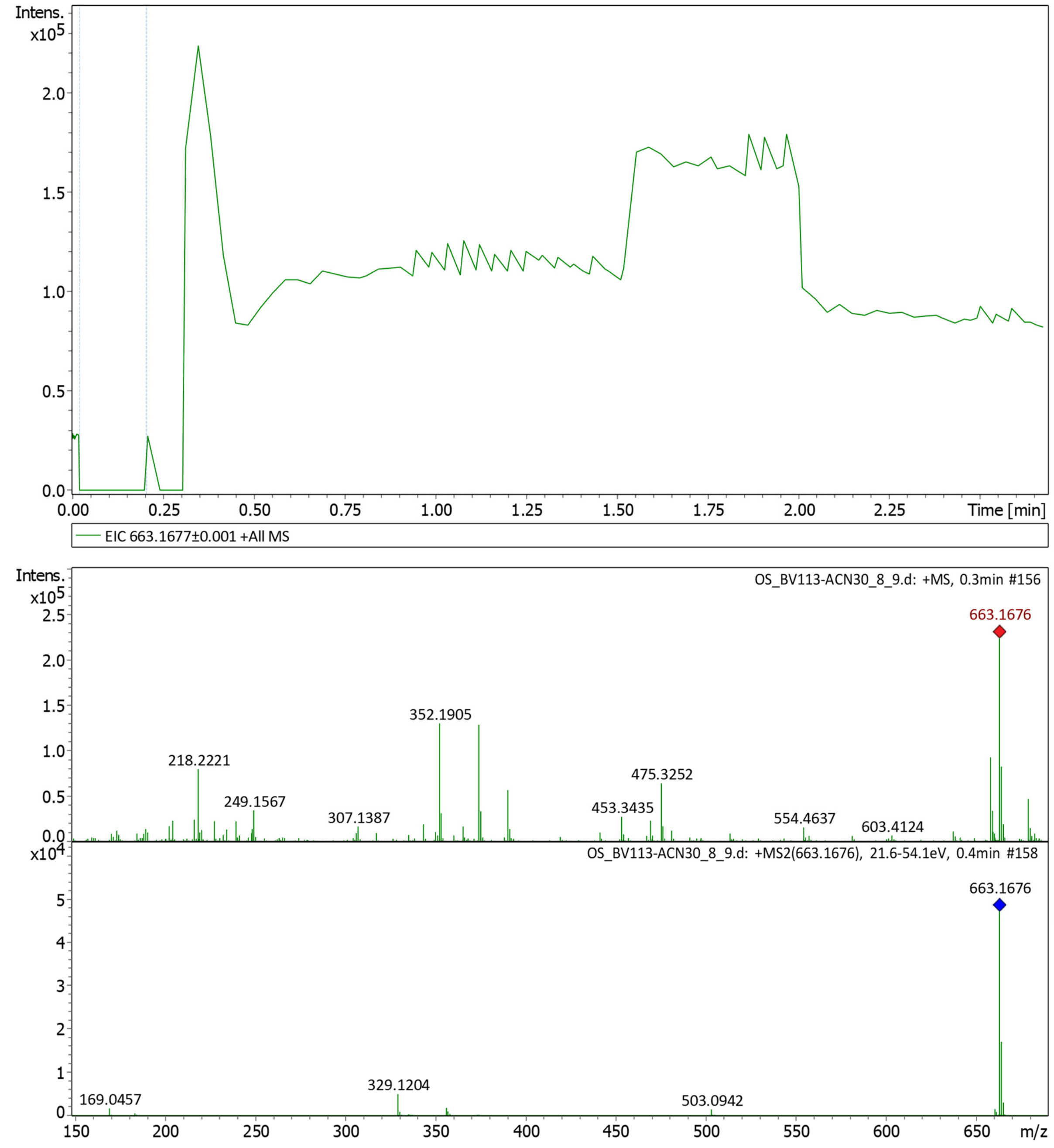

The data aquired with direct sample injection, no retention time included in this report.

Table S1. Physiological and biochemical characteristics of KB-1 isolate and closely related *Streptomyces* sp. strains

**Properties**	**KB-1**	***Streptomyces zaomyceticus*** **NRRL B-2038^T**	***Streptomyces exfoliatus*** **NBRC 13191^T**	***Streptomyces venezuelae*** **ATCC 10712^T**
Cultural characteristics				
Growth on ISP2/ISP3/ISP4	Good	Good	Good	Good
Color of aerial mycelium on ISP2/ISP3/ISP4	White/light gray/ light gray	White/gray/gray	White/rose/rose	Gray/white/gray
Colony Reverse on ISP2/ISP3/ISP4	Colorless	Brown/ivory/ivory	Ivory/yellow/gray brown	Brown/yellow/ivory
Diffusible pigment on ISP2/ISP3/ISP4	None	None	None	Brown/none/none
Melanoid pigment on ISP6	—	+	—	+
Morphological characteristics				
Spore surface	Smooth	Smooth	Smooth	Smooth
Spore chains morphology	Straight	Straight	Straight	Straight
Physiological characteristics				
Temperature-range and optimum (°C)	15-30 (25)	(30)	(30)	(30)
Acid from carbohydrates				
Arabinose	—	+	+	+
Fructose	—	+/-	+	+/-
Galactose	—	n/d	n/d	n/d
Glucose	+	+	+	+
Inositol	—	+/-	—	—
Maltose	+	n/d	n/d	n/d
Mannitol	—	+/-	—	—
Raffinose	—	—	+	—
Rhamnose	—	—	+	+/-
Sorbitol	—	n/d	n/d	n/d
Sucrose	—	+/-	+	—
Xylose	—	+/-	+	—
Degradation of				
Starch	+	n/d	n/d	n/d
Gelatin	+	+	n/d	+

Casein	+	n/d	n/d	n/d
Cellulose	++	—	n/d	—

+, positive; —, negative; n/d, not determined

The sources used for: *Streptomyces zaomyceticus* NRRL B-2038^T and *Streptomyces exfoliatus* NBRC 13191^T [1], *Streptomyces venezuelae* ATCC 10712^T (https://bacdive.dsmz.de (accessed on 11 April 2022)).

Table S2. Physiological and biochemical characteristics of BV113 isolate and closely related *Streptomyces* sp. strains

Properties	BV113	*Streptomyces osmaniensis* OU-63^T	*Streptomyces galbus* JCM 4570^T	*Streptomyces chartreusis* NBRC 12753^T
Cultural characteristics				
Growth on ISP2/ISP3/ISP4	Good	Good	Good	Good
Color of aerial mycelium on ISP2/ISP3/ISP4	White/Pale green/ pale blue	Bluish gray	Silk gray/traffic gray/ light ivory	Pastel turquoise
Colony reverse on ISP2/ISP3/ISP4	None/yellow/none	Pale yellow	Orange brown/honey yellow/ochre brown	Colorless
Diffusible pigment on ISP2/ISP3/ISP4	None/yellow/none	None	Honey yellow/lemon yellow/honey yellow	None
Melanoid pigment on ISP6	+	+	—	+
Morphological characteristics				
Spore surface	Spiny	Spiny	Smooth	Spiny
Spore chains morphology	Spirales	Spirales	Spirales	Spirales
Physiological characteristics				
Temperature-range and optimum (°C)	20-40 (37)	15-40	(28)	(30)
pH-range and optimum	5-9 (7)	6-10	n/d	n/d
Acid from carbohydrates				
Arabinose	+	+	+	+
Fructose	+	+	+	+
Galactose	+	+	n/d	+
Glucose	+	+	+	+
Inositol	—	+	+	+
Maltose	—	+	n/d	n/d
Mannitol	+	+	n/d	+
Raffinose	+	+	+	+
Rhamnose	+	+	—	+
Sorbitol	—	+	n/d	n/d
Sucrose	+	+	+	+
Xylose	—	+	+	+
Degradation of				
Starch	+	+	n/d	+
Gelatin	+	+	+	+

Casein	+	—	n/d	+
Cellulose	+	+	+	+
Antibiotic resistance				
Erythromycin, 5 µg/disk	resistant	n/d	n/d	n/d
Vancomycin, 5 µg/disk	sensitive	n/d	n/d	n/d
Levofloxacin, 5 µg/disk	sensitive	n/d	n/d	n/d
Tetracycline, 12.5 µg/disk	sensitive	n/d	n/d	n/d
Streptomycin, 10 µg/disk	sensitive	sensitive	n/d	n/d

+, positive; —, negative; n/d, not determined

The sources used for: *Streptomyces osmaniensis* OU-63^{T} [2], *Streptomyces galbus* JCM 4570^{T} and *Streptomyces chartreusis* NBRC 12753^{T} (https://bacdive.dsmz.de (accessed on 11 April 2022)).

Table S3. Composition of some nutrient media used

Medium	Acronym	Ingredients (g/L)	References
Mineral agar Gauze 1	G1	Soluble starch - 20<br>KNO_3 - 1<br>$K_2HPO_4 \cdot 7H_2O$ - 0.5<br>NaCl - 0.5<br>$MgSO_4 \cdot 7H_2O$ - 0.5<br>$FeSO_4 \cdot 7H_2O$ - 0.01<br>pH adjusted to 7.2<br>Agar - 20	[3]
Organic medium 79	Org79	Glucose - 10<br>Peptone - 10<br>Casein hydrolysate - 2<br>Yeast extract - 2<br>NaCl - 6<br>pH adjusted to 7.0<br>Agar - 20	[4]
Glucose-asparagine agar	GA	Glucose - 10<br>Asparagine - 0.5<br>K_2HPO_4 - 0.5<br>Agar - 20	[5]
Soy flour mannitol agar	SFM	Mannitol - 20<br>Soy flour - 20<br>pH adjusted to 8.0<br>Agar - 20	[6]
Yeast extract-malt extract agar	ISP2	Bacto-yeast extract - 4<br>Bacto-malt extract - 10<br>Bacto-dextrose - 4<br>pH adjusted to 7.3<br>Bacto-agar - 20	[7]
Oatmeal agar	ISP3	Oatmeal - 20<br>$FeSO_4 \cdot 7H_2O$ - 0.001<br>$MnCl_2 \cdot 4H_2O$ - 0.001<br>$ZnSO_4 \cdot 7H_2O$ - 0.001<br>pH adjusted to 7.2<br>Agar - 18	[7]
Inorganic salts-starch agar	ISP4	Soluble starch - 10<br>K_2HPO_4 - 1<br>$MgSO_4 \cdot 7H_2O$ - 1<br>NaCl - 1<br>$(NH_4)_2SO_4$ - 2<br>$CaCO_3$ - 2<br>$FeSO_4 \cdot 7H_2O$ - 0.001<br>$MnCl_2 \cdot 4H_2O$ - 0.001	[7]

		$ZnSO_4 \cdot 7H_2O$ - 0.001<br>pH adjusted to 7.0 - 7.4<br>Agar - 20	
Peptone-yeast extract iron agar	ISP6	Bacto-peptone iron agar - 36<br>Bacto-yeast extract - 1<br>pH adjusted to 7.0 - 7.2	[7]
Basal agar medium	ISP9	Carbon source - 1% (w/v)<br>$(NH_4)_2SO_4$ - 2.64<br>KH_2PO_4 - 2.38<br>$K_2HPO_4 \cdot 3H_2O$ - 5.65<br>$MgSO_4 \cdot 7H_2O$ - 1<br>$CuSO_4 \cdot 5H_2O$ - 0.0064<br>$FeSO_4 \cdot 7H_2O$ - 0.0011<br>$MnCl_2 \cdot 4H_2O$ - 0.0079<br>$ZnSO_4 \cdot 7H_2O$ - 0.0015<br>pH adjusted to 6.8 - 7.0<br>Agar - 15	[7]
Actinomyces Isolation Agar	M490	Sodium caseinate - 2<br>L-Asparagine - 0.1<br>Sodium propionate - 4<br>K_2HPO_4 - 0.5<br>$MgSO_4$ - 0.1<br>$FeSO_4$ - 0.001<br>pH adjusted to 8.1 ± 0.2<br>Agar - 15<br>Glycerol - 5 ml	[8]<br>(HiMedia Laboratories)
Humic acid-vitamin agar	HV agar	Humic acid - 1<br>Na_2HPO_4 - 0.5<br>KCl - 1.71<br>$MgSO_4 \cdot 7H_2O$ - 0.05<br>$FeSO_4 \cdot 7H_2O$ - 0.01<br>$CaCO_3$ - 0.02<br>Thiamine-HCl - 0.0005<br>Riboflavin - 0.0005<br>Niacin - 0.0005<br>Pyridoxine-HCl - 0.0005<br>Inositol - 0.0005<br>Ca-pantothenate - 0.0005<br>*p*-aminobenzoic acid - 0.0005<br>Biotin - 0.00025<br>pH adjusted to 7.2<br>Agar - 18	[9]

Supplementary References

1. Shirling, E.B.; Gottlieb, D. Cooperative description of type cultures of Streptomyces. II. Species descriptions from first study. *International Journal of Systematic Bacteriology* **1968**, *18*, 69-189, doi:10.1099/00207713-18-2-69.
2. Reddy, T.V.K.; Mahmood, S.; Idris, M.M.; Ciesielski, S. *Streptomyces osmaniensis* sp. nov., isolated from soil. *International journal of systematic and evolutionary microbiology* **2010**, *60*, 1755-1759, doi:10.1099/ijs.0.017749-0.
3. Gause, G.F.; Preobrazhenskaya, T.P.; Sveshnikova, M.A.; Terekhova, L.P.; Maksimova, T.S. *Guide for Determination of Actinomycetes: Genera Streptomyces, Streptoverticillium, and Chainia.*; Nauka: Moscow, USSR, 1983; Volume 1.
4. Prauser, H.; Falta, R. [Phage sensitivity, cell wall composition and taxonomy of actinomyctes]. *Zeitschrift fur allgemeine Mikrobiologie* **1968**, *8*, 39-46, doi:10.1002/jobm.3630080106.
5. Krainsky, A. Die Actinomyceten und ihre Bedeutung in der Natur. *Zentralblatt fur Bakteriologie, Parasitenkunde, Infektionskrankheiten und Hygiene. 1. Abt. Medizinisch-hygienische Bakteriologie, Virusforschung und Parasitologie. Originale* **1914**, *42*, 649-688.
6. Hobbs, G.; Frazer, C.M.; Gardner, D.C.; Cullum, J.A.; Oliver, S.G. Dispersed growth of Streptomyces in liquid culture. *Applied Microbiology and Biotechnology* **1989**, *31*, 272-277, doi:10.1007/BF00258408.
7. Shirling, E.B.; Gottlieb, D. Methods for characterization of *Streptomyces* species. *International Journal of Systematic Bacteriology* **1966**, *16*, 313-340, doi:10.1099/00207713-16-3-313.
8. MacFaddin, J.F. *Media for Isolation-Cultivation-Identification-Maintenance of Medical Bacteria*; Williams & Wilkins: Baltimore, MD, USA, 1985; Volume 1.
9. Hayakawa, M.; Nonomura, H. Humic Acid-Vitamin Agar, a New Medium for the Selective Isolation of Soil Actinomycetes. *Journal of Fermentation Technology* **1987**, *65*, 501-509, doi:10.1016/0385-6380(87)90108-7.
